# Pennsylvania Hospital

**Published:** 1842-01-01

**Authors:** 


### CLINICAL REPORTS.

### Pennsylvania Hospital—*Surgical Wards—Service of* Dr. E. Peace.

### *Large doses of Iodide of Potassium in Scrofula.*

1. J. B., negro, æt. 25, admitted March 11, for scrofulous ulceration of the throat internally, attended with great hoarseness. Various astringent gargles were employed in succession, the ulcers were frequently cauterised with solid nitrate of silver, and an alterative course of treatment instituted.

This was continued six months without permanent benefit; for, although during this period the patient occasionally improved under cauterisation and the daily thorough application of some astringent lotion, yet he as often relapsed, until, in the end, he appeared to be nearly as hoarse as ever. Half a fluid-drachm of the iodide of potassium in at least two fluid ounces of water, twice a day, was then, (Sept. 13,) prescribed, and all the previous remedies, general and local, were omitted. The dose was gradually increased to half a fluid drachm four times in the day.

No unpleasant symptoms were excited by the operation of the medicine. The improvement was immediate, well marked, and rapid. It steadily advanced, until he became so well satisfied of his permanent restoration that he solicited his discharge, left the hospital November 3, almost well, and is now employed at sea.

2. G. W., æt. 21, was admitted Oct. 25, complaining of severe osteocopic pains, particularly in the left tibia,—on which there was a small venereal node,—and of intense lancinating pain in the head. On account of the disease of the left tibia, the patient was unable to walk without difficulty and pain. Some months previously, he had contracted primary syphilis, for which he had been freely salivated. Ordered a blistering plaster to the node, a warm bath in the evening to be repeated every other night, and half a drachm of the iodide of potassium in a pint of water to be taken in the course of the day.

The iodide was omitted the next day, on account of some febrile symptoms, and under the apprehension of aggravating the cephalalgia by it under the circumstances. The patient was then purged, restricted in diet, and depleted locally by means of cups over the temples, behind the ears, and at the back of the neck; and, subsequently, by means of leeches freely applied over the forehead; but with very transient alleviation of the headach, and no abatement of the pains in the extremities.

After an interval of three or four days, the iodide was resumed in doses of seven and a half grains, with half a fluid ounce of syr. sarsap. diluted with a little water, four times daily. The patient's diet was improved, he was directed to walk about in the open air, and an anodyne was administered every night. This plan was steadily pursued, the dose of the iodide being gradually increased until it amounted to fifty-two grains in the twenty-four hours. The headach soon began to diminish, and within a week had so far subsided as to render the anodyne at night unnecessary. The pains in the limbs ceased, the node on the tibia disappeared by degrees, and the patient slowly recovered the entire use of his left leg. He was discharged November 22, rapidly convalescing; continued to take the iodide and the syrup sarsap. according to direction about eight days longer, and is now entirely well.

